# The Role of Computer Skills in Personal Health Record Adoption Among Patients With Heart Disease: Multidimensional Evaluation of Users Versus Nonusers

**DOI:** 10.2196/19191

**Published:** 2021-07-26

**Authors:** Martina A Clarke, Ann L Fruhling, Elizabeth L Lyden, Alvin E Tarrell, Tamara L Bernard, John R Windle

**Affiliations:** 1 School of Interdisciplinary Informatics College of Information Science and Technology University of Nebraska Omaha Omaha, NE United States; 2 Division of Cardiovascular Medicine Department of Internal Medicine University of Nebraska Medical Center Omaha, NE United States; 3 Department of Biostatistics College of Public Health University of Nebraska Medical Center Omaha, NE United States

**Keywords:** patient portal, communication, patients, precision medicine, health literacy

## Abstract

**Background:**

In the era of precision medicine, it is critical for health communication efforts to prioritize personal health record (PHR) adoption.

**Objective:**

The objective of this study was to describe the characteristics of patients with heart disease that choose to adopt a PHR.

**Methods:**

A total of 79 patients with chronic cardiovascular disease participated in this study: 48 PHR users and 31 nonusers. They completed 5 surveys related to their choice to use or not use the PHR: demographics, patient activation, medication adherence, health literacy, and computer self-efficacy (CSE).

**Results:**

There was a significant difference between users and nonusers in the sociodemographic measure education (*P*=.04). There was no significant difference between users and nonusers in other sociodemographic measures: age (*P*=.20), sex (*P*=.35), ethnicity (*P*=.43), race (*P*=.42), and employment (*P*=.63). There was a significant difference between PHR users and PHR nonusers in CSE (*P*=.006).

**Conclusions:**

In this study, we demonstrate that sociodemographic characteristics were not an important factor in patients’ use of their PHR, except for education. This study had a small sample size and may not have been large enough to detect differences between groups. Our results did demonstrate that there is a difference between PHR users and nonusers related to their CSE. This work suggests that incorporating CSE into the design of PHRs is critical. The design of patient-facing tools must take into account patients’ preferences and abilities when developing effective user-friendly health information technologies.

## Introduction

### Precision Medicine in Cardiovascular Disease

The Precision Medicine Initiative is a nationwide initiative that was launched in 2015 to transform the United States health care delivery from a “one-size-fits-all” approach to one that takes into account individual variability in genes, environment, and lifestyle when providing patients with treatment and prevention strategies [1]. The primary goal of precision medicine is to provide optimized medical care and outcomes for each patient. Benefits of precision medicine include increased prediction capabilities to determine which treatments work best for particular patients; better insight into the underlying mechanisms by which multiple diseases occur; enhanced methods for preventing, diagnosing, and treating a variety of illnesses; and improved electronic health records (EHRs) integration in clinical care, which promotes easier access to health data [2].

Management of life-threatening illnesses and chronic diseases has been progressing toward precision medicine for many years [3]. There has been exceptional precision medicine advancement related to cardiovascular disease (CVD) [4-8], which aids in the transformation of the management practice for CVD. Despite these advancements, CVD still ranks as one of the leading causes of death in the United States [9]. CVD contributes US $320 billion to health care costs annually. This includes the cost of health care services, medicines, and lost productivity due to death. This cost is projected to increase to US $818 billion by 2030 [10]. In addition to focusing on preventive measures to reduce the incidence of CVD, improving current patients’ management of this disease will reduce the CVD’s economic cost on the health care system. Although targeted therapies related to cardiovascular medicine are less developed than in other specialties, such as oncology, these therapies have been acknowledged as a practical next step in patient-centered CVD treatments [4]. Patient-centered care relies heavily on patients becoming more involved in their health and wellness in order to achieve the optimal benefits from the health care system. Effective communication between health care providers and patients is necessary for health promotion efforts to be successful. New strategies, such as the personal health record (PHR), have been implemented to enhance the effectiveness of health information communication between patients and their providers.

### Patient Portal Use

The Medicare and Medicaid EHR Incentive Programs advocate for patients to be involved in their health care. The PHR allows patients to electronically view their health information, after-visit summaries, credible educational materials, and reconcile their medication list [11]. PHR use should improve patient–provider communication, self-management of chronic illnesses, and medication adherence [12,13]. However, a data brief from the Office of the National Coordinator for Health Information Technology reported that although more than 90% of health care organizations offer patient portal access, less than 25% of patients actually use it [14]. Another study by Powell and Deroche [15] found that 35% of patients with a chronic disease have never used their patient portal. Patients with a chronic illness play an important role in their health improvement because chronic illnesses require continuous self-management efforts [16-19]. A study by Henry et al [20] found that providing educational information in patient portals can improve chronic disease self-management. Patients managing a chronic illness must be involved in and knowledgeable about their health. Patients who are engaged in their care are more inclined to follow treatment plans and manage their health [14,21,22].

Precision medicine offers promising improvements to health care. However, for this potential to materialize, it is necessary to involve patients in the process. In addition to generating targeted therapies, precision medicine will also generate complex risk and benefit information that will be hard to interpret for low-literacy populations [23]. Adoption of precision medicine in CVD will require patients to interact with complex results in their PHR. A literature review by Wynn et al [24] found that a patient’s health literacy impacts his/her ability to understand precision medicine materials; therefore, providing patient-facing materials that are understandable to all health literacy levels must be a priority when designing health information technology (HIT) tools.

Educational gaps in precision medicine exist for patients, which requires interventions to be implemented to improve knowledge, awareness, and attitude on how precision medicine will be incorporated into the patient experience and the PHR [25]. For patients to receive the most value from their PHR, the information presented within must be written at a level comprehensible to a lay audience so that they have the ability to act on the information received [26-30]. Research is needed to determine appropriate data display, visual aids, and understandable language that will foster adoption of the PHR; however, evidence remains limited in this area [24,31]. Previous research has focused on patient portal use among patients with multiple chronic illnesses, but none have focused solely on patients with CVD.

### Objective

The promotion of technology-assisted disease self-management is increasing as PHRs continue to be adopted by health care organizations. Therefore, the objective of this study is to describe the characteristics of patients with heart disease who choose to adopt PHRs. Sociodemographic and propensity characteristics were explored among PHR users and non-PHR users.

## Methods

### Study Design

This study involved multiple, previously validated surveys completed by cardiovascular medicine patients affiliated with the University of Nebraska Medical Center (UNMC). This survey was administered between August 2015 and June 2019. UNMC’s Institutional Review Board approved this study as an expedited research project.

### Organizational Setting

UNMC is an academic medical center whose clinical partner is Nebraska Medicine. The Division of Cardiovascular Medicine operates 3 clinics with over 28,000 annual patient visits. The team includes experts in general cardiology and a team of leading subspecialists in areas such as cardiac electrophysiology, interventional cardiology, structural heart disease, diagnostic cardiovascular imaging, congenital heart disease, advanced heart failure, mechanical circulatory support, and heart transplants. A nonprofit organization, Healthcare Information and Management Systems Society, rated UNMC with Stage 7 of the Electronic Medical Record (EMR) Adoption Model in 2016 [32]. A Stage 7 rating is awarded to hospitals and clinics with a fully integrated EHR that transports data using Continuity of Care Documents, utilizes data warehousing to assess clinical data, and demonstrates summary data continuity for all hospital services [33]. The PHR offered at UNMC is Epic (Verona) MyChart, a tethered PHR, and was available to patients at the beginning of 2014.

### Recruitment

For our study, we recruited patients who received care at the UNMC’s Heart and Vascular Center. When eligible patients were identified, a nurse coordinator contacted patients via a telephone call. The recruitment phone call introduced the voluntary nature of the study, and explained what the patient’s participation in study would entail. Data collection sessions were scheduled and conducted in a clinic or adjacent conference room. Whenever feasible, the data collection session was linked to patients’ scheduled appointment for convenience. This method of connecting the data collection session with patients’ upcoming clinic visit was especially appealing to busier young and middle-aged adults. Participants were not compensated for their participation.

### Participants

Overall, recruitment response was positive. A total of 95 patients were screened for participation in this research project. Of those, 16 declined while 79 accepted and participated in the research. Eligible participants were current patients of UNMC, scheduled for a clinic visit follow-up, 19 years old and older, and able to give consent. Use of the PHR was not a screening criterion. PHR users were defined as research patients who signed up for Nebraska Medicine’s Epic MyChart and sending at least one message prior to enrollment in this research project. Of the 79 who participated in the research there were 48 users and 31 non-users of the PHR.

### Data Collection

Each data collection session lasted 15-30 minutes. After consent was obtained, the survey was administered. Sociodemographic data were collected followed by administration of 5 survey tools: the Computer Self-Efficacy (CSE) Survey, the Health Literacy Survey, the Medication Adherence Survey (MAS), and the Patient Activation Measure (PAM). These battery of surveys measure the patient’s comfort using computers, their general medical knowledge, their likelihood of taking prescribed medications, and their engagement in their care.

### Measures

#### Computer Self-Efficacy

The CSE questionnaire is a 10-item survey that utilizes an 11-point Likert scale, and asks the patients their confidence in completing a task under a variety of scenarios, such as when given step-by-step instructions, utilizing on-call user help, or initial training in getting started. Scores for each question range between 0 and 10, with the total score then being between 0 and 100. The CSE has long been used to assess users’ belief that they can successfully interact with a computer system. Based on social psychology, self-efficacy has been found to influence the users’ behavior related to their use of the system [34].

#### Health Literacy Survey

The Health Literacy Survey is a 3-item survey that measures patients’ adequacy in understanding health information. Developed and validated by Chew et al [35,36], the Health Literacy Scale works well in a busy clinical environment. Health literacy and PAMs are both correlated with health outcomes, however Smith et al [37] noted a poor correlation between the 2 measures and argued that both should be targeted to improve patient safety and engagement.

#### Medication Adherence Survey

MAS is an 8-item patient survey that provides reliable predictions of patient medication compliance [38]. MAS has a strong correlation with clinical outcomes in patients with hypertension and other conditions [39]. Patients with greater knowledge, attitude, satisfaction, and coping skills were more likely to have high medication adherence, whereas those stressed or requiring a complex medication scheme were less likely to be adherent [40].

#### Patient Activation Measure

The PAM (Insignia Health) is a 13-item survey using a 4-point Likert scale. It is a robust and well-validated assessment tool developed by Hibbard and colleagues [41] to measure the level of patients’ engagement in their health. The PAM scale reflects a developmental model of activation. Activation appears to involve 4 stages: (1) believing the patient role is important, (2) having the confidence and knowledge necessary to take action, (3) actually taking action to maintain and improve one’s health, and (4) staying the course even under stress.

### Data Analysis

Survey data were recorded and stored in a secure database and analyzed using SAS 9.4 (SAS Institute) in conjunction with a biostatistician (EL). The data were summarized using descriptive statistics which included counts and percentages, means, SDs, medians, and interquartile ranges. Patient characteristics were compared between PHR users and non-users using the Fisher exact test for categorical data and the 2-sample unpaired *t* test for continuous data. The 2-sample *t* test was used to compare the composite scores for the survey instruments between the groups. Missing data were handled using pairwise deletion (available-case analysis). In other words, results were reported for the nonmissing values for each variable analyzed. All tests were 2-sided and a *P* value of <.05 was considered statistically significant.

## Results

Table 1 shows the demographics of patients with CVD that participated in this study. Responses are classified according to PHR users (48 participants) and PHR nonusers (31 participants). There was a significant difference between users and nonusers in the sociodemographic measure education (*P*=.04). There was no significant difference between users and nonusers in the sociodemographic measures age (*P*=.17), sex (*P*=.35), ethnicity (*P*=.43), race (*P*=.42), and employment (*P*=.75).

There was a significant difference (*P*=.006) between PHR users and PHR nonusers in CSE (Table 2).

**Figure 1:**
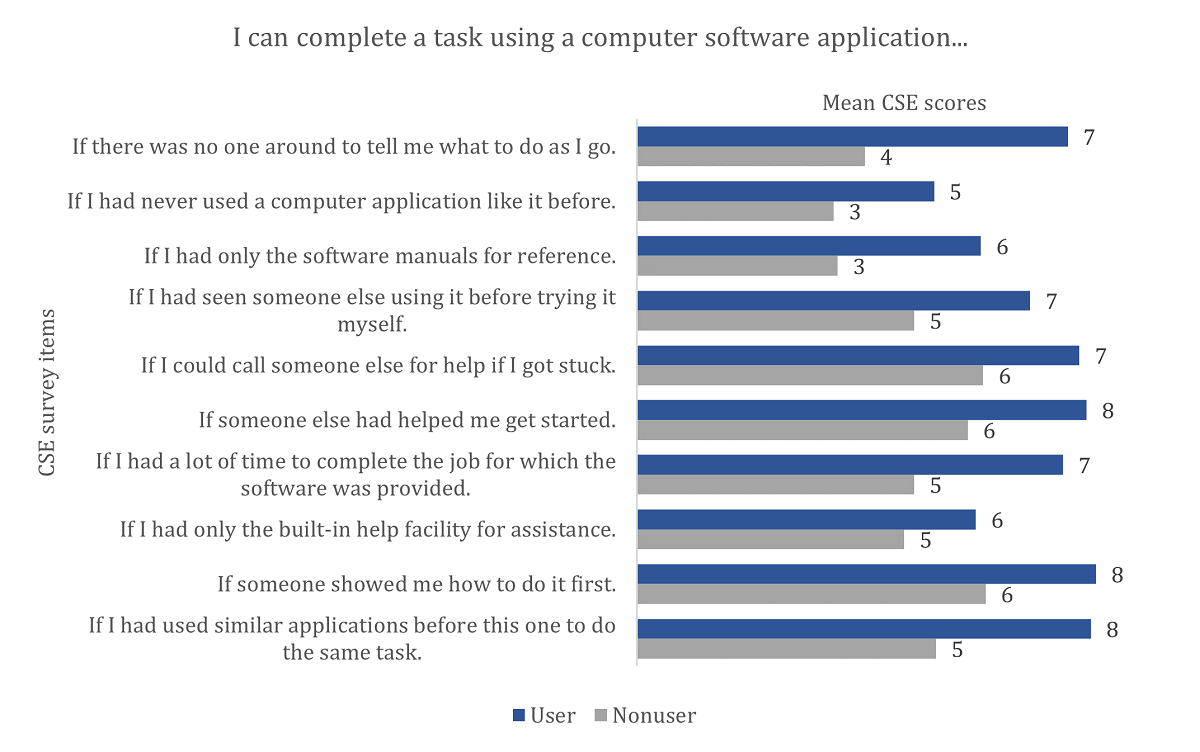
Mean CSE scores by survey items for PHR users and nonusers. Scores range from 0 to 10. CSE: computer self-efficacy; PHR: personal health record.

Figure 1 shows the mean CSE scores by survey items for PHR users and nonusers. Both users and nonusers reported being less able to complete a task using a computer software application if they had never used a computer application like it before.

**Table 1:** Distribution of population characteristics categorized by PHR users and PHR nonusers (N=79).

Demographics		Nonuser (n=31)	User (n=48)	*P* value
Age (years), mean		63	57	.20
**Sex, n (%)**				.36
	Male	18 (58)	22 (46)	
	Female	13 (42)	26 (54)	
**Education, n (%)**				.04
	Some high school	3 (10)	1 (2)	
	High-school graduate/general educational diploma	9 (29)	9 (19)	
	Some college/associate degree	10 (32)	17 (35)	
	College graduate	8 (26)	9 (19)	
	Postsecondary education	1 (3)	12 (25)	
**Ethnicity, n (%)**				.43
	Hispanic/Latino	4 (13)	7 (15)	
	Not Hispanic or Latino	27 (87)	41 (85)	
**Race, n (%)**				.42
	American Indian/Alaskan Native	1 (3)	0 (0)	
	Black/African American	6 (19)	6 (13)	
	White	24 (77)	42 (88)	
**Employment, n (%)**				.63
	Employed	11 (35)	19 (40)	
	Unemployed	20 (65)	29 (60)	

**Table 2:** Characteristics of PHR users and PHR nonusers.

Characteristics	Nonuser				User			
	N	Mean (SD)	Median (IQR)	N		Mean (SD)	Median (IQR)	*P* value
Computer Self-Efficacy	30	46.23 (34)	45.5 (65)	48		66.58 (28.95)	73.50 (45.50)	.006
Health Literacy	31	8.42 (29)	8 (2)	48		8.25 (1.3)	8 (2)	.72
Medication Adherence	15	6.87 (1.25)	7 (2.25)	23		6.28 (1.4)	7 (1.25)	.20
Patient Activation	31	64.34 (17.8)	55.62 (21.6)	48		67.45 (18)	67.82 (21.1)	.50
Patient Activation Level	31	2.61 (1.05)	3 (2)	48		3 (0.9)	3 (1)	.08

## Discussion

### Principal Findings

#### Overview

Our results show a significant difference between PHR users and PHR nonusers’ education (*P*=.04) and CSE (*P*=.006). This study adds to the ongoing discussion about the adoption of PHRs with a focus on patients with CVD. However, these results cannot be generalized to patients with an acute illness because of the different care required from chronic diseases. Although chronic disease is a major health care issue in the United States, the health care system is slow to re-adjust from an acute care focus to a system that addresses the complexities of chronic disease [42]. The use of HIT, such as PHRs, can support the management of chronic diseases. There is a lack of studies that look at PHR adoption among patients with heart disease. Patients with heart disease are an important group to study because CVD is a complex chronic disease and is one of the leading causes of death in the United States. Heart disease and stroke account for almost 801,000 deaths annually, costing US $316 billion in health care expenditures and lost productivity annually [43]. CSE plays a role in PHR acceptance and use. Previous literature on PHR adoption shows a difference between race and ethnicity in PHR use. Multiple studies found that Black and Hispanic patients were less likely to use a PHR as well as individuals with Medicare or Medicaid insurance [44-49]. Most of those studies recruited based on specific demographics as dependent not independent variables. Our results suggest that awareness of these disparities may have bolstered strategies focused on the demographics with lower usage rates in an effort to increase adoption [50]. A deeper analysis is needed to validate these results.

#### CSE’s Role in PHR Use

Our results demonstrate a difference between PHR users and nonusers in CSE, but not in other measured scales. CSE is significantly influenced by one’s computer knowledge and previous computer experience [51]. Having prior computer knowledge before using a PHR would increase CSE scores and likelihood of PHR use. Patients are more open to trying a PHR because they are familiar with how computer applications work.

#### Patient Activation Was Not a Factor in PHR Adoption

Another interesting finding is that patient activation was not a factor in PHR adoption. Previous studies have shown an association between PHR use and improved levels of patient activation [52-54]. Patients who are engaged in their care are less likely to adopt the PHR if they also have low self-efficacy. Our results implicate that patient’s comfort using technology plays a more important role in PHR use than patient activation.

#### Recommendations

It is important to address the challenges in using HIT for patients with lower literacy levels [55]. It is critical that the PHR’s display be tailored to the comprehension abilities of individuals with low computer literacy. Further, technological support should be made available when possible. Future research should examine the feasibility of computerized adaptive tests as screening tools to identify patients’ literacy skills [56,57]. Future research should also compare characteristics of patients receiving acute care versus chronic care in terms of their PHR adoption and use. Understanding these differences will assist in developing targeted interventions to improve PHR use.

### Limitations

This study was limited to patients with cardiology issues; therefore, this study needs to be reproduced in other practice settings because of the specific information needs of the different specialties [58,59]. Recruitment came from a single academic medical center and the results need to be validated in multiple academic centers. This study had a small sample size and may not have been large enough to detect differences between groups. There may be specific patient characteristics that were not measured in this cohort that may have an effect on patients’ PHR usage.

### Conclusions

CSE played a role in whether or not a patient would be a PHR user. Design of patient-facing tools must take into account patients’ preferences and abilities when developing effective user-friendly HIT tools [60]. Providing tools designed for the “average patient” will result in isolation of patients that do not fit into the “average” mold. Future research should explore the PHR features most used by patients with cardiology issues to understand how to prioritize functionality. Future HIT tools should be developed to overcome gaps in CSE. PHRs have the promise of improving chronic disease management and increasing patient engagement. Optimizing the PHR to support its intended users will provide the momentum needed to increase patient engagement in their care [61-63].

## Abbreviations

CSE: computer self-efficacy
CVD: cardiovascular disease
EHR: electronic health record
EMR: Electronic Medical Record
HIT: health information technologies
MAS: Medication Adherence Survey
PAM: Patient Activation Measure
PHR: personal health record
UNMC: University of Nebraska Medical Center

## References

[1] Office of the Press Secretary (2015). FACT SHEET: President Obama's Precision Medicine Initiative. Statements & Releases, 2015.

[2] Genetics Home Reference What are some potential benefits of precision medicine and the Precision Medicine Initiative? Help Me Understand Genetics: Precision Medicine 2020 March 17, 2020. MedlinePlus Genetics.

[3] Rosen S (2017). Why Precision Medicine Continues to Be the Future of Health Care. Oncology Times.

[4] Antman EM, Loscalzo J (2016). Precision medicine in cardiology. Nat Rev Cardiol.

[5] Costantino S, Libby Peter, Kishore Raj, Tardif Jean-Claude, El-Osta Assam, Paneni Francesco (2018). Epigenetics and precision medicine in cardiovascular patients: from basic concepts to the clinical arena. Eur Heart J.

[6] Currie G, Delles Christian (2018). Precision Medicine and Personalized Medicine in Cardiovascular Disease. Adv Exp Med Biol.

[7] Fatkin D, Huttner IG, Kovacic JC, Seidman J, Seidman CE (2019). Precision Medicine in the Management of Dilated Cardiomyopathy: JACC State-of-the-Art Review. J Am Coll Cardiol.

[8] Shah SJ (2017). Precision Medicine for Heart Failure with Preserved Ejection Fraction: An Overview. J Cardiovasc Transl Res.

[9] Heron M (2019). Deaths: Leading Causes for 2017. Natl Vital Stat Rep.

[10] Giedrimiene D, King R (2017). Abstract 207: Burden of Cardiovascular Disease (CVD) on Economic Cost. Comparison of Outcomes in US and Europe. Circ: Cardiovascular Quality and Outcomes.

[11] Fricton JR, Davies D, Henriksen Kerm, Battles James, Keyes Margaret, Grady Mary (2008). Personal Health Records to Improve Health Information Exchange and Patient Safety. Advances in Patient Safety: New Directions and Alternative Approaches (Vol. 1: Assessment).

[12] Kruse CS, Argueta DA, Lopez L, Nair A (2015). Patient and provider attitudes toward the use of patient portals for the management of chronic disease: a systematic review. J Med Internet Res.

[13] The Office of the National Coordinator for Health Information Technology (ONC) (2014). Step 5: Achieve Meaningful Use Stage 2 Monday, February 24.

[14] Henry JaWanna, Barker Wes, Kachay Lolita (2019). Electronic Capabilities for Patient Engagement among U.S. Non-Federal Acute Care Hospitals: 2013-2017. ONC Data Brief.

[15] Powell KR, Deroche C (2020). Predictors and patterns of portal use in patients with multiple chronic conditions. Chronic Illn.

[16] Barello S, Graffigna G, Vegni E (2012). Patient engagement as an emerging challenge for healthcare services: mapping the literature. Nurs Res Pract.

[17] James J (2013). Patient engagement. Health Affairs Health Policy Brief. 14(10.1377).

[18] Stewart MT, Hogan TP, Nicklas J, Robinson SA, Purington CM, Miller CJ, Vimalananda VG, Connolly SL, Wolfe HL, Nazi KM, Netherton D, Shimada SL (2020). The Promise of Patient Portals for Individuals Living With Chronic Illness: Qualitative Study Identifying Pathways of Patient Engagement. J Med Internet Res.

[19] Clarke MA, Moore JL, Steege LM, Koopman RJ, Belden JL, Canfield SM, Kim MS (2018). Toward a patient-centered ambulatory after-visit summary: Identifying primary care patients' information needs. Inform Health Soc Care.

[20] Henry SL, Shen E, Ahuja A, Gould MK, Kanter MH (2016). The Online Personal Action Plan: A Tool to Transform Patient-Enabled Preventive and Chronic Care. Am J Prev Med.

[21] Malhotra A, Crocker ME, Willes L, Kelly C, Lynch S, Benjafield AV (2018). Patient Engagement Using New Technology to Improve Adherence to Positive Airway Pressure Therapy: A Retrospective Analysis. Chest.

[22] Painter J, Moore B, Morris G (2015). Addressing medication non-adherence through implementation of an appointment-based medication synchronization network.

[23] Kutner M (2006). The Health Literacy of America's Adults: Results from the 2003 National Assessment of Adult Literacy.

[24] Wynn RM, Adams KT, Kowalski RL, Shivega WG, Ratwani RM, Miller KE (2018). The Patient in Precision Medicine: A Systematic Review Examining Evaluations of Patient-Facing Materials. J Healthc Eng.

[25] Lee M, Flammer AJ, Lerman LO, Lerman A (2012). Personalized medicine in cardiovascular diseases. Korean Circ J.

[26] Berland GK, Elliott MN, Morales LS, Algazy JI, Kravitz RL, Broder MS, Kanouse DE, Muñoz J A, Puyol J, Lara M, Watkins KE, Yang H, McGlynn EA (2001). Health information on the Internet: accessibility, quality, and readability in English and Spanish. JAMA.

[27] Eysenbach G, Powell J, Kuss O, Sa E (2002). Empirical studies assessing the quality of health information for consumers on the world wide web: a systematic review. JAMA.

[28] Kasabwala K, Agarwal N, Hansberry DR, Baredes S, Eloy JA (2012). Readability assessment of patient education materials from the American Academy of Otolaryngology--Head and Neck Surgery Foundation. Otolaryngol Head Neck Surg.

[29] Tang PC, Ash JS, Bates DW, Overhage JM, Sands DZ (2006). Personal Health Records: Definitions, Benefits, and Strategies for Overcoming Barriers to Adoption. Journal of the American Medical Informatics Association.

[30] Clarke MA, Fruhling AL, Sitorius M, Windle TA, Bernard TL, Windle JR (2020). Impact of Age on Patients' Communication and Technology Preferences in the Era of Meaningful Use: Mixed Methods Study. J Med Internet Res.

[31] Clarke MA, Schuetzler RM, Windle JR, Pachunka E, Fruhling A (2020). Usability and cognitive load in the design of a personal health record. Health Policy and Technology.

[32] (2016). Stage 7 Case in Point: Nebraska Medicine.

[33] (2014). Electronic Medical Record Adoption Model (EMRAM).

[34] Compeau DR, Higgins CA (1995). Computer Self-Efficacy: Development of a Measure and Initial Test. MIS Quarterly.

[35] Chew LD, Bradley KA, Boyko EJ (2004). Brief questions to identify patients with inadequate health literacy. Fam Med.

[36] Chew LD, Griffin JM, Partin MR, Noorbaloochi S, Grill JP, Snyder A, Bradley KA, Nugent SM, Baines AD, Vanryn Michelle (2008). Validation of screening questions for limited health literacy in a large VA outpatient population. J Gen Intern Med.

[37] Smith SG, Curtis LM, Wardle J, von Wagner C, Wolf MS (2013). Skill set or mind set? Associations between health literacy, patient activation and health. PLoS One.

[38] Morisky DE, Ang A, Krousel-Wood M, Ward HJ (2008). Predictive validity of a medication adherence measure in an outpatient setting. J Clin Hypertens (Greenwich).

[39] Lavsa SM, Holzworth A, Ansani NT (2011). Selection of a validated scale for measuring medication adherence. Journal of the American Pharmacists Association.

[40] Hibbard JH, Mahoney E (2010). Toward a theory of patient and consumer activation. Patient Educ Couns.

[41] Hibbard JH, Mahoney ER, Stockard J, Tusler M (2005). Development and testing of a short form of the patient activation measure. Health Serv Res.

[42] Murrow EJ, Oglesby FM (1996). Acute and Chronic Illness. Orthopaedic Nursing.

[43] Benjamin Emelia J, Blaha Michael J, Chiuve Stephanie E, Cushman Mary, Das Sandeep R, Deo Rajat, de Ferranti Sarah D, Floyd James, Fornage Myriam, Gillespie Cathleen, Isasi Carmen R, Jiménez Monik C, Jordan Lori Chaffin, Judd Suzanne E, Lackland Daniel, Lichtman Judith H, Lisabeth Lynda, Liu Simin, Longenecker Chris T, Mackey Rachel H, Matsushita Kunihiro, Mozaffarian Dariush, Mussolino Michael E, Nasir Khurram, Neumar Robert W, Palaniappan Latha, Pandey Dilip K, Thiagarajan Ravi R, Reeves Mathew J, Ritchey Matthew, Rodriguez Carlos J, Roth Gregory A, Rosamond Wayne D, Sasson Comilla, Towfighi Amytis, Tsao Connie W, Turner Melanie B, Virani Salim S, Voeks Jenifer H, Willey Joshua Z, Wilkins John T, Wu Jason Hy, Alger Heather M, Wong Sally S, Muntner Paul, American Heart Association Statistics CommitteeStroke Statistics Subcommittee (2017). Heart Disease and Stroke Statistics-2017 Update: A Report From the American Heart Association. Circulation.

[44] Spooner KK, Salemi JL, Salihu HM, Zoorob RJ (2016). Disparities in perceived patient-provider communication quality in the United States: Trends and correlates. Patient Educ Couns.

[45] Lustria MLA, Smith SA, Hinnant CC (2011). Exploring digital divides: an examination of eHealth technology use in health information seeking, communication and personal health information management in the USA. Health Informatics J.

[46] Beckjord EB, Finney Rutten LJ, Squiers L, Arora NK, Volckmann L, Moser RP, Hesse BW (2007). Use of the internet to communicate with health care providers in the United States: estimates from the 2003 and 2005 Health Information National Trends Surveys (HINTS). J Med Internet Res.

[47] Jung C, Padman R, Shevchik G, Paone S (2011). Who are portal users vs. early e-Visit adopters? A preliminary analysis. AMIA Annu Symp Proc.

[48] Anthony DL, Campos-Castillo C, Lim PS (2018). Who Isn't Using Patient Portals And Why? Evidence And Implications From A National Sample Of US Adults. Health Aff (Millwood).

[49] Turner K, Hong Young-Rock, Yadav Sandhya, Huo Jinhai, Mainous Arch G (2019). Patient portal utilization: before and after stage 2 electronic health record meaningful use. J Am Med Inform Assoc.

[50] Fraccaro P, Vigo Markel, Balatsoukas Panagiotis, Buchan Iain E, Peek Niels, van der Veer Sabine N (2017). Patient Portal Adoption Rates: A Systematic Literature Review and Meta-Analysis. Stud Health Technol Inform.

[51] John S (2013). Influence of computer self-efficacy on information technology adoption. International Journal of Information Technology.

[52] Kipping S, Stuckey MI, Hernandez A, Nguyen T, Riahi S (2016). A Web-Based Patient Portal for Mental Health Care: Benefits Evaluation. J Med Internet Res.

[53] Schnock KO, Snyder JE, Fuller TE, Duckworth M, Grant M, Yoon C, Lipsitz S, Dalal AK, Bates DW, Dykes PC (2019). Acute Care Patient Portal Intervention: Portal Use and Patient Activation. J Med Internet Res.

[54] Nagykaldi Z, Aspy CB, Chou A, Mold JW (2012). Impact of a Wellness Portal on the delivery of patient-centered preventive care. J Am Board Fam Med.

[55] Kim H, Xie B (2017). Health literacy in the eHealth era: A systematic review of the literature. Patient Educ Couns.

[56] Rose M, Bjorner Jakob B, Fischer Felix, Anatchkova Milena, Gandek Barbara, Klapp Burghard F, Ware John E (2012). Computerized adaptive testing--ready for ambulatory monitoring?. Psychosom Med.

[57] Hsueh PS, Zhu X, Ramakrishnan S (2014). Progressive testing of health self-efficacy and literacy for personalized engagement. Stud Health Technol Inform.

[58] Collins SA, Currie LM, Bakken S, Cimino JJ (2009). Information needs, Infobutton Manager use, and satisfaction by clinician type: a case study. J Am Med Inform Assoc.

[59] Currie L, Graham Mark, Allen Mureen, Bakken Suzanne, Patel Vimla, Cimino James J (2003). Clinical information needs in context: an observational study of clinicians while using a clinical information system. AMIA Annu Symp Proc.

[60] Demiris G (2016). Consumer Health Informatics: Past, Present, and Future of a Rapidly Evolving Domain. Yearb Med Inform.

[61] Keselman A, Slaughter Laura, Smith Catherine Arnott, Kim Hyeoneui, Divita Guy, Browne Allen, Tsai Christopher, Zeng-Treitler Qing (2007). Towards consumer-friendly PHRs: patients' experience with reviewing their health records. AMIA Annu Symp Proc.

[62] Haga SB, Mills R, Pollak KI, Rehder C, Buchanan AH, Lipkus IM, Crow JH, Datto M (2014). Developing patient-friendly genetic and genomic test reports: formats to promote patient engagement and understanding. Genome Med.

[63] Hulse NC, Ranade-Kharkar P, Post H, Wood GM, Williams MS, Haug PJ (2011). Development and early usage patterns of a consumer-facing family health history tool. AMIA Annu Symp Proc.

